# Uterus didelphys with unilateral obstructed hemivagina with hematometrocolpos and hematosalpinx with ipsilateral renal agenesis

**DOI:** 10.4103/0974-1208.57230

**Published:** 2009

**Authors:** Gaurav Jindal, Satish Kachhawa, G L Meena, Gopal Dhakar

**Affiliations:** Department of Radiodiagnosis, Sardar Patel Medical College, Bikaner, Rajasthan - 334 001, India

**Keywords:** Renal agenesis, unilateral hematometrocolpos, uterus didelphys

## Abstract

Uterus didelphys with blind hemivagina and ipsilateral renal agenesis (Herlyn Werner-Wunderlich Syndrome) is a rare congenital anomaly. It mostly presents with severe dysmenorrhea and a palpable mass due to unilateral hematocolpos. A patient with dysmenorrhea from a double uterus and an obstructed hemivagina is a diagnostic dilemma because the menses are regular. We report a case of a 14-year-old girl with this condition who was diagnosed as uterus didelphys with unilateral hematocolpos and hydrosalpinx with ipsilateral renal agenesis on the basis of sonography and confirmed by laparoscopic examination.

## INTRODUCTION

The association of uterus didelphys with unilateral obstructed hemivagina with hematometrocolpos and hematosalpinx with ipsilateral renal agenesis has been recognized. Sonography shows characteristic imaging findings, which we found in a 14- year- old girl presenting with dymenorrhea along with a progressively enlarging pelvic mass associated with normal menstrual cycles.

## CASE REPORT

A 14-year-old girl was admitted to our institute with complaints of recurrent pelvic pain, mainly at the time of menses, and a gradually increasing lower abdominal swelling since menarche, which was attained at the age of 12 years. Her menstrual cycles were regular and flow was adequate.

General physical examination was unremarkable except for mild pallor. Per abdominal examination revealed a well-defined cystic mass arising from the pelvis.

**Figure 1:**
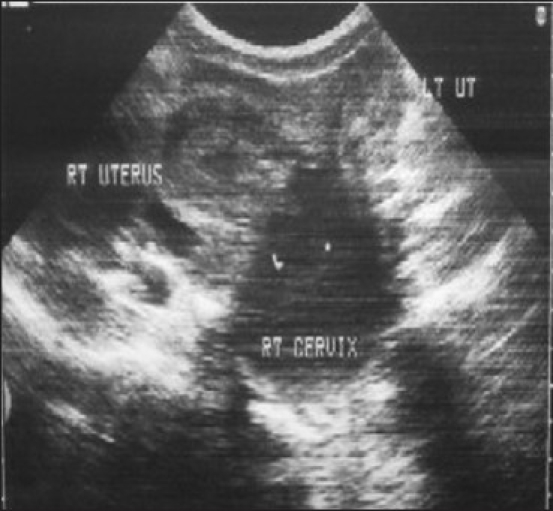
Transabdominal ultrasound showing two widely divergent uterine horns with no communication between them and a distended right cervix

**Figure 2:**
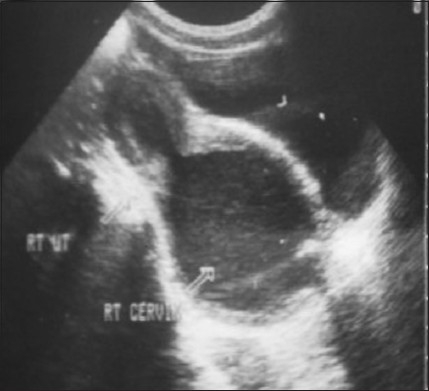
Transabdominal USG showing right endometrial cavity distended with fluid containing low-level internal echoes and grossly distended right cervix

**Figure 3:**
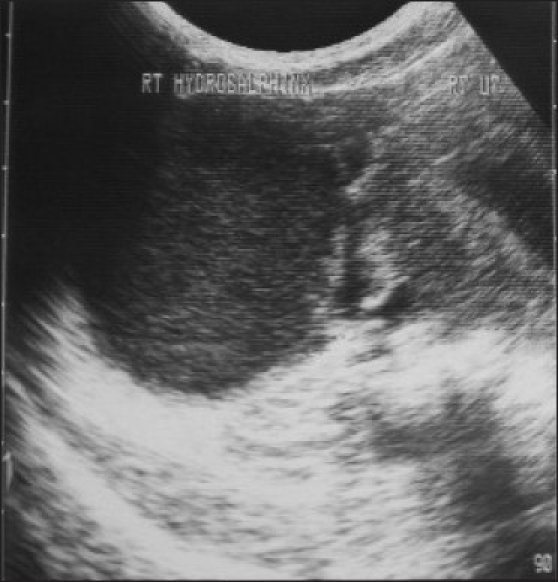
A large tubular cystic mass is seen adjacent to the right uterus, confirmed on laparoscopy to be a hydrosalpinx

The patient was referred to our department for ultrasonography of abdomen and pelvis (USG), which showed two widely divergent uterine horns with no communication between them [Figure 1]. The right endometrial cavity was distended with fluid containing low-level internal echoes and grossly distended right cervix [Figure 2]. The left cervix was not clearly visualized as it was possibly compressed by the distended right cervix. A large tubular cystic mass was seen adjacent to the right uterus, possibly suggestive of hydrosalpinx [Figure 3].

Ultrasonography of abdomen and pelvic region revealed an empty right renal fossa. On the basis of imaging findings, a diagnosis of uterus didelphys with unilateral obstructed hemivagina with resultant hematometrocolpos and hydrosalpinx with ipsilateral renal agenesis was made.

Vaginal examination under anesthesia showed a large bulge high up on the right lateral wall. A vaginal septotomy was given and 200 mL of chocolate-colored fluid was drained. Subsequent USG revealed normal-sized bilateral uterine cavities and cervices. The cystic pelvic mass however persisted. The patient underwent laparoscopy, which confirmed the diagnosis, and laparoscopic drainage of the hydrosalpinx was performed.

## DISCUSSION

The uterus, fallopian tubes, cervix and upper 2/3^rd^s of the vagina develop from the paired mullerian ducts while the lower 1/3^rd^ of the vagina develops separately from the urogenital sinus. The association of uterus didelphys with obstructed hemivagina with renal agenesis ipsilateral to the side of obstruction can be explained by embryologic arrest at the 8^th^ gestation week, which simultaneously affects the mullerian and metanephric ducts. Certain other renal anomalies may also be associated, such as renal dysplasia, double collecting system and ectopic ureter.[1] The condition has also been reported to be associated with high-riding aortic bifurcation, Inferior vena cava (IVC) duplication, intestinal malrotation and ovarian malposition.[2]

Mullerian duct anomalies (MDA) are congenital anomalies of the female genital tract that result from nondevelopment or nonfusion of the mullerian ducts or failed resorption of the uterine septum.[3,5]

Incidence of these anomalies is believed to be between 0.5 and 5.0%.[6,7]

Majority of these cases are diagnosed at menarche. Early and accurate diagnosis is vital because untreated cases may develop retrograde tubal reflux and endometriosis. It may also cause impaired fertility and obstetric complications later in life.

MDAs are divided into six groups on the basis of the Buttram and Gibbons system.[8]

**Table:** 

Class I:	Uterine/cervical hypoplasia or agenesis (very rare).
Class II:	Nicornuate uterus (15%) due to partial/complete agenesis.
Class III:	Uterine didelphys due to complete failure of fusion of the two mullerian ducts resulting in complete duplication anomaly with formation of two widely divergent uterine horns and two cervices.
Class IV:	Bicornuate uterus due to partial failure of mullerian duct fusion.
Class V:	Septate or subseptate uterus due to partial or complete failure of resorption of the midline septum after normal mullerian duct fusion.
Class VI:	Uterine anomalies resulting from *in utero* exposure to diethylstilbestrol (DES), characterized as a T-shaped uterus.

The specific association of uterus didelphys, obstructed hemivagina with unilateral hematocolpos and ipsilateral renal agenesis is well recognized.[1] The obstructing vaginal septum seen in this condition is usually oblique/ longitudinal and varies in thickness from very thin to quite thick. In transverse vaginal septum, a vertical fusion disorder exists between the Müllerian ducts and the urogenital sinus. It may be complete or incomplete and is not usually associated with other urologic or Müllerian anomalies.[9] It presents with cyclic abdominal pain after menarche associated with progressively enlarging pelvic mass but normal menstrual cycles. Distal obstruction may result in reversed menstruation with the development of right hematosalpinx, such as in our case.

Resection of the vaginal septum is the treatment of choice for obstructed hemivagina. In cases where the obstructed hemivagina reaches the hymeneal ring, a limited resection– marsupialization and insertion of a Foley's catheter may be performed during an initial surgical procedure, allowing the remaining vaginal septum to be removed later.[10] Alternately, hysteroscopic resection of the septum under transabdominal ultrasouind guidance may also be carried out, especially in young females, so as to preserve hymenal integrity.[1]

Imaging modalities used to diagnose this condition include ultrasonography, conventional and sonohysterosalpingography and magnetic resonance imaging (MRI). Computed tomography (CT) has a limited role in evaluation of the female pelvis, although the latest three-dimensional multiplanar CTs can diagnose this condition and associated urinary anomalies, it is mostly not preferred because of radiation exposure. Ultrasound is a cheap, noninvasive, widely available imaging modality that accurately helps in the diagnosis of this condition. The vaginal septum however is difficult to visualize on ultrasound and is best shown on MRI.
